# Root microbiome diversity and structure of the Sonoran desert buffelgrass (*Pennisetum ciliare* L.)

**DOI:** 10.1371/journal.pone.0285978

**Published:** 2023-05-19

**Authors:** Angélica Jara-Servin, Adán Silva, Hugo Barajas, Rocío Cruz-Ortega, Clara Tinoco-Ojanguren, Luis D. Alcaraz

**Affiliations:** 1 Laboratorio de Genómica Ambiental, Departamento de Biología Celular, Facultad de Ciencias, Universidad Nacional Autónoma de México, Mexico City, Mexico; 2 Posgrado en Ciencias Bioquímicas, Universidad Nacional Autónoma de México, Mexico City, Mexico; 3 Departamento de Ecología de la Biodiversidad, Instituto de Ecología, Universidad Nacional Autónoma de México, Hermosillo, Sonora, Mexico; 4 Departamento de Ecología Funcional, Instituto de Ecología, Universidad Nacional Autónoma de México, Mexico City, Mexico; Universidade Federal de Minas Gerais, BRAZIL

## Abstract

Buffelgrass (*Pennisetum ciliare*) is an invasive plant introduced into Mexico’s Sonoran desert for cattle grazing and has converted large areas of native thorn scrub. One of the invasion mechanisms buffelgrass uses to invade is allelopathy, which consists of the production and secretion of allelochemicals that exert adverse effects on other plants’ growth. The plant microbiome also plays a vital role in establishing invasive plants and host growth and development. However, little is known about the buffelgrass root-associated bacteria and the effects of allelochemicals on the microbiome. We used 16S rRNA gene amplicon sequencing to obtain the microbiome of buffelgrass and compare it between samples treated with root exacknudates and aqueous leachates as allelochemical exposure and samples without allelopathic exposure in two different periods. The Shannon diversity values were between *H’* = 5.1811–5.5709, with 2,164 reported bacterial Amplicon Sequence Variants (ASVs). A total of 24 phyla were found in the buffelgrass microbiome, predominantly *Actinobacteria*, *Proteobacteria*, and *Acidobacteria*. At the genus level, 30 different genera comprised the buffelgrass core microbiome. Our results show that buffelgrass recruits microorganisms capable of thriving under allelochemical conditions and may be able to metabolize them (*e*.*g*., *Planctomicrobium*, *Aurantimonas*, and *Tellurimicrobium*). We also found that the community composition of the microbiome changes depending on the developmental state of buffelgrass (*p* = 0.0366; ANOSIM). These findings provide new insights into the role of the microbiome in the establishment of invasive plant species and offer potential targets for developing strategies to control buffelgrass invasion.

## 1. Introduction

Introducing exotic species to a new environment can alter the ecosystem and decrease biological diversity. Invasive species can transform ecosystem processes over spatial and temporal scales, with various degrees of impact [1]. In many cases, invasive species have higher values of resource-acquisition traits, larger sizes, and higher growth rates, implying larger pool sizes of nutrients that allow them to outcompete native species [1]. Buffelgrass (*Pennisetum ciliare*, *Cenchrus ciliaris*) has been North America’s most noxious invasive plant since its introduction from East Africa in the 1930s [2]. The germination, establishment, and subsequent seedling growth of buffelgrass depend on soil moisture and environmental temperature conditions [3]. Buffelgrass is preferred because of its high germinability, easy establishment, high seedling production and vigor, fast growth rate, and ability to withstand fires because of its massive root system [3–6]. Buffelgrass individual tussocks and their long lifespan allow them to re-sprout from established tufts following a fire [5]. Fire also temporarily increases available phosphorus levels in the soil, which are rapidly exploited by buffelgrass [7].

In northwestern Mexico, large tracts of desert and thorn scrub have been converted to buffelgrass pasture to improve rangelands for cattle production [8]. Buffelgrass coverage estimates are 53% of the Sonora state and 12% of Mexico [9]. In the Sonoran Desert, buffelgrass invasion can cause a decrease by 53%–73% of the number of native plant species compared to undisturbed thorn scrub, which has a higher species diversity and is commonly dominated by Fabaceae and Cactaceae [8]. In contrast, thorny legume trees such as *Acacia cochliacantha* and *Acacia farnesiana* dominate the succession of abandoned pastures in the thorn scrub of Mexico [10], and very few native species can regenerate in the ranges [8]. The approaches taken to control buffelgrass growth include applying herbicides, manual removal, prescribed burning, and controlled animal grazing, but controlling buffelgrass worldwide is still an urgent matter [5].

One of the mechanisms proposed as a strategy for invasive species control is allelopathy [8, 11], which is based on the active synthesis and release of allelochemicals (secondary metabolites) by one plant, influencing the growth of a recipient plant, regardless of resource availability [12, 13]. The production of biochemicals that natives in the invaded range do not produce plays in favor of the invasive species since these chemicals may affect native species that lack a coevolutionary-based tolerance [14, 15]. Allelopathy plays a vital role in invasion, vegetation patterning, the exclusion of associated species, and reduced plant productivity [16]. Previous reports have shown buffelgrass allelopathy through root exudates of 3-month-old individuals, decreasing the growth and germination of various species such as *Chrysopogon aucheri*, *Hyparrhenia rufa*, *Bothriochloa pertusa*, *Panicum antidotale*, *Setaria italica*, and *Pennisetum americanum* [4]. Buffelgrass leachates and root exudates obtained from 3-month-old buffelgrass plants and used to water *Brassica campestris*, *Lactuca sativa*, and *Setaria italica* provoked a reduction in radicle growth due to allelopathy [17]. The germination rates of these three species also decreased, but only when the leachates and exudates were concentrated through evaporation [18]. The decrease in wild species could result from the amount of forage buffelgrass produced, which is 4 to 10 times greater than the production of native species, leading to possible nutrient depletion, especially of N, from the soils it grows on [3]. Such characteristics may explain, to a certain degree, the observed self-declination of pastures of *Pennisetum ciliare* [3, 18].

Various approaches have been made to describe the chemical identity of buffelgrass allelochemicals affecting the growth of many plant species. So far, the identified compounds are phenolic compounds, including *p-*OH-benzoic acid, *p-*coumaric acid, caffeic acid, vanillic acid, ferulic acid, syringic acid, and gentisic acid [17]. Although phenolic compounds have a short half-life in soils [19, 20], their reversibly sorbed fractions contribute to the pool available for allelopathic interactions [19]. Hence, compounds such as ferulic acid, *p*-coumaric acid, *p*-OH-benzoic acid, and vanillic acid could accumulate in the soil and impede the germination and growth of susceptible species [17]. However, the fate and persistence of phytotoxins in soil are unclear, as are their phytotoxicity or biodisposition by microbes [17]. The mutualistic association formed by the invader is presumed to contribute to a competitive advantage over native species [21, 22], and plants can impact their associated microbiomes as an adaptation strategy when confronted with biotic and abiotic challenges [15, 23, 24]. Moreover, allelopathic plants release compounds that might alter the composition of the microbial community, recruiting microorganisms involved in the metabolization of the allelochemicals produced [25, 26]. Hence, understanding the rhizosphere microbiomes of invasive plants and the elements that influence the recruitment of microorganisms could be particularly valuable for understanding the factors promoting plant invasiveness and the following impacts on the ecosystems. So far, there are no reports of the buffelgrass rhizosphere microbiome composition and the effects of its allelochemicals on the microbial communities associated with its roots.

This work investigated the buffelgrass root microbiome and its compositional changes under allelochemical exposure. Microbiome changes were tested at two-time points using plants exposed to buffelgrass root exudates, watered with leachates from the aerial part of buffelgrass, and with a distilled water regime. This experimental design allowed us to describe the buffelgrass microbiome throughout buffelgrass growth, obtain a core microbiome, and determine the effect that allelochemicals may have on taxa comprising the rhizosphere microbiome.

## 2. Methods

### 2.1 Seed and soil sampling

*Pennisetum ciliare* seeds were collected from five plants at the end of the summer (September 2013) from induced pastures at Rancho Diamante (28° 41’ N, 110° 15’ W), Sonora State, Mexico, where thorn scrub is the original vegetation. Seeds were stored in a paper bag and transported to the laboratory. We also collected soil samples from the same location for further plant growth in the greenhouse. We collected samples of Buffelgrass *Cenchrus ciliaris* (*Penisetum ciliare*) from a location where we did not require special permissions, beyond the land owner. The land owner permitted us to collect the samples. It is important to note that while Buffelgrass is native to Tropical Africa and Asia, it is considered an invasive species in North America. However, it is not classified as an endangered or protected species and, therefore, does not require special permission for collection.

### 2.2 Plant growth under allelopathic conditions

**Fig 1:**
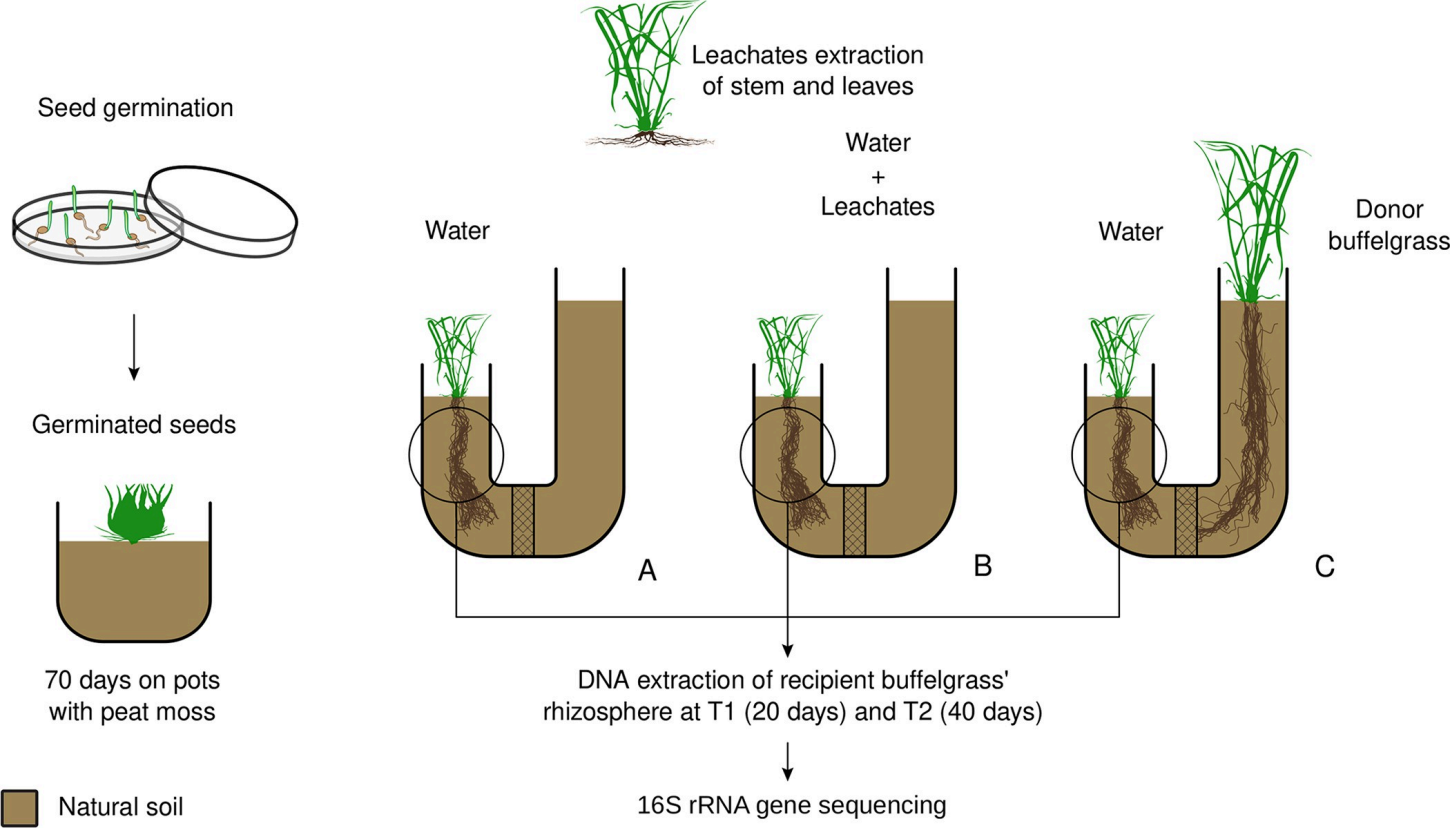
Experimental setup. Buffelgrass seeds germinated in a chamber and, after two days, transferred to peat moss-filled pots. After 70 days, the plants were transplanted into PVC pots containing silica and soil (1:1) from Rancho Diamante, Sonoran Desert. The plants were then divided into three groups, each with four replicates. Group (A) was watered with sterilized distilled water, Group (B) was watered with a solution of aqueous leachates from leaves and stems, and Group (C) were watered with distilled water but exposed to root exudates from another buffelgrass plant. Sampling was done at two different periods, 20 and 40 days, with two replicates from each group being analyzed at each time.

We removed the seed covers of *P*. *ciliare* seeds for germination as naked caryopses have higher and more uniform germination [27]. Caryopses were germinated in Petri dishes with 2% agar in a growth chamber Biotronette (20.3L, 25°C, 12-h photoperiod, 92 μmolm^-2^s^-1^). Subsequently, 2-day-old seedlings were transferred to containers with peat moss, which grew for 70 days. The water regime consisted of watering with Hoagland solution every two weeks. After that, 70-day-old plants with a height of at least 10 cm were transplanted into curved PVC tubes containing sterilized silica sand and soil (at a proportion of 1:1) from Rancho Diamante. Plants in PVC tubes received a standard water regime (consisting of 25 mL of sterilized deionized water six times per week). They were divided into three different treatments: (1) root exudates, (2) aqueous leachates of the aerial plant part, and (3) control (Fig 1, https://doi.org/10.6084/m9.figshare.c.6605350.v2). The exudate treatment consisted of a second buffelgrass plant at the opposite end of the curved PVC tube, so the roots of the 70-day-old individual would be under the influence of the second buffelgrass plant, allowing to evaluate the effect of the root exudates on the rhizosphere microbiome. Physical interaction of the roots was avoided by installing a net at the middle of the tube. In the leachate treatment, one end of the PVC tube was planted with the 70-day-old buffelgrass, while the other was left unplanted. Plants under this treatment were watered with aqueous leachates extracted from buffelgrass green leaves and stems instead of water, but according to the volume and periodicity of the standard water regime. The control treatment consisted of only one end of the PVC planted with buffelgrass with the standard water regime. No Hoagland solution was added during the allelochemical treatments. The leachates were obtained as previously described [28] by collecting green leaves and stems of buffelgrass adult plants in Rancho El Diamante. Leaves were dried for 24 h at 24°C and submerged in distilled water 1% (g/v) for 3 h, followed by filtering through Whatman #4 filtering paper to simulate rainfall. Two soil samples from Rancho Diamante were left unplanted and subjected to no treatment. These soil samples allowed us to determine the soil microbiome of the region without the influence of growing buffelgrass plants and the effect that buffelgrass has on the recruited microorganisms.

### 2.3 Rhizosphere sample collection

Rhizosphere samples were obtained at two different time points of growth from the PVC tubes: after 20 days (T1) and after 40 days (T2). In both cases, the roots were separated from the remaining parts of the plant to obtain the rhizosphere. Rhizosphere separation involved removing the loose soil and subsequently washing and submitting the roots to ultrasound in 1X PBS buffer (137 mM NaCl; 2.7 mM KCl; 10 mM Na_2_HPO_4_; 1.8 mM KH_2_PO_4_). All pellets obtained from buffelgrass rhizospheres were recovered through centrifugation (50-mL tubes, 1,300 g, 10 min) and kept at -80°C until further analysis and DNA extraction.

### 2.4 Metagenomic DNA processing and 16S rRNA gene sequencing

The metagenomic DNA of soil samples and all the rhizosphere pellets was extracted using the Mobio PowerSoil DNA extraction kit (MoBio, Carlsbad, CA, United States) following the manufacturer’s instructions, with minor modifications, heating the C6 elution solution to 60°C before the elution step to increase the DNA yield. Two 30 μL elution processes were performed during the same spin filter.

The 16S rRNA gene amplification was performed in duplicates, following the Illumina ® MiSeq protocol for 16S metagenomic sequencing library preparation (Illumina 2013). The primer pair used for the PCR amplification was 341F/805R (targeting the V3–V4 regions), with the Illumina sequencing adaptors in 5′ overhangs (341F: 5′ -CCTACGGGNGGCWGCAG-3′; 805R: 5′ - ACTACHVGGGTATCTAATCC 3′). The PCR reaction mixture consisted of 2 μL buffer, 1.2 μL of each primer (5 μM), 3 μL enhancer, 1.6 μL dNTPs (2.5 mM), 0.6 μL Mg2S04 (1.5μM), 9.2 μL PCR-grade water, 0.16 μL Pfx polymerase (0.02 U/μL) (Invitrogen, Thermo Fisher Scientific, Waltham, MA), and 2 μL DNA template, adding up to a final volume of 20 μL.

The PCR conditions were as follows: initial 95°C for 3 min, then 25 cycles at 94°C for 5 s, followed by 68°C for 30 s. The PCR products were pooled and purified with the SV Wizard PCR purification kit (Promega, Madison, WI). All samples were sequenced on the Illumina ® MiSeq platform (2 × 300 paired-end) at the University Unit of Massive Sequencing and Bioinformatics Facilities of the Biotechnology Institute, UNAM Mexico.

### 2.5 16S rRNA gene amplicon sequence analysis

The detailed protocol and bioinformatic methods used to process and analyze the 16S rRNA gene amplicon sequences are available on GitHub (https://github.com/genomica-fciencias-unam/buffelgrass). Briefly, all amplicon libraries were quality-checked using Dada2 [29], the first 17 bp were removed, and the sequences were trimmed to 250 bp. Only the forward reads were used since the quality profiles of the reverse reads were poor. All amplicon sequences were processed to obtain Amplicon Sequence Variants (ASV) using DADA2 (v. 1.10.1) [29] to denoise and remove chimeras. At the species level, taxonomy was assigned using the Silva database (v. 138) [30]. A phylogenetic tree was constructed using FastTreeMP [31] (S1 Dataset, https://doi.org/10.6084/m9.figshare.c.6605350.v2).

### 2.6 Diversity and statistical analysis

The α- and β-diversity of all samples were calculated using the phyloseq [32], ggplot2 [33], vegan [34], and R default packages [35]. We measured taxonomic α-diversity using Observed, Shannon, and Simpson diversity indices. Hierarchical clustering was performed using the hclust method on an unweighted UniFrac distance matrix [36]. The ASVs were clustered at the various taxonomic levels to perform abundance comparisons of the three treatments. Core microbiomes were analyzed using the upset function; ß-diversity was analyzed through a constrained analysis of principal coordinates (CAP) on an unweighted UniFrac distance matrix, based on the obtained ASV abundances per treatment; the clustering was evaluated through the ANOSIM statistical function [37]. Differential ASV abundances comparing treatments were calculated using DESeq2 of the R package [38]. Detailed statistical and bioinformatic methods are available on FigShare (https://doi.org/10.6084/m9.figshare.c.6605350.v2).

## 3. Results

We sequenced 686,078 paired-end reads, with a mean of 49,005.57 ± 10,041 sequences per sample (n = 8). We then clustered them into 2,164 Amplicon Sequence Variants (ASVs; 100% identity 16S rRNA gene OTUs) and described diversity by Observed ASVs, Simpson, and Shannon diversity indices (Table 1). The Shannon index (*H’*) was used to evaluate the **α**-diversity of our samples. Overall, the samples maintained a similar diversity (*H’* = 5.1811–5.5709), regardless of treatment or period, except for the outliers, corresponding to samples of the leachate treatment at the first period (*H’* = 4.4335 and *H’* = 5.8719). The diversity increased slightly with the development of buffelgrass treated with exudates (from *H’* = 5.2984 and 5.4687 in the first period to *H’* = 5.5709 and 5.4640 in the second one). On the contrary, control samples showed a diversity reduction as the experiment advanced (*H’* = 5.3508 and 5.4666 in the first period to *H’* = 5.2681 and 5.2257 in the second one). Species dominance, evaluated through the Simpson index (*D*), followed the same pattern as the one described for diversity, where the value for the exudate treatment was higher at the end of the experiment (*D* = 0.9947 and 0.9945) than at the beginning (*D* = 0.9932 and 0.9943), as opposed to the increase seen from the first to the second period in samples from the control treatment (*D* = 0.9934 and 0.9942 to *D* = 0.9935 and 0.9928). Soil samples had Shannon diversity (*H’* = 5.361425 and 5.331116) and Simpson (*D* = 0.9938688 and 0.9935597) indices not differing from the treatment values.

**Table 1:** Alpha diversity for buffelgrass microbiomes.

Sample	Observed	Shannon	Simpson
**C1_T1**	308	5.350832	0.9934471
**C2_T1**	349	5.466695	0.9942502
**C1_T2**	270	5.268196	0.9935561
**C2_T2**	262	5.225775	0.9928567
**E1_T1**	291	5.298459	0.9932077
**E2_T1**	342	5.468742	0.9943344
**E1_T2**	384	5.570910	0.9947818
**E2_T2**	332	5.464093	0.9945279
**L1_T1**	126	4.433554	0.9849328
**L2_T1**	538	5.871980	0.9957321
**L1_T2**	262	5.181120	0.9923309
**L2_T2**	282	5.291607	0.9935689
**S1**	297	5.361435	0.9938688
**S2**	295	5.331116	0.9935597

Values calculated for Observed, Shannon, and Simpson diversity indices. C is for controls; E exudates; L leachate; S soil; T is for time.

**Fig 2:**
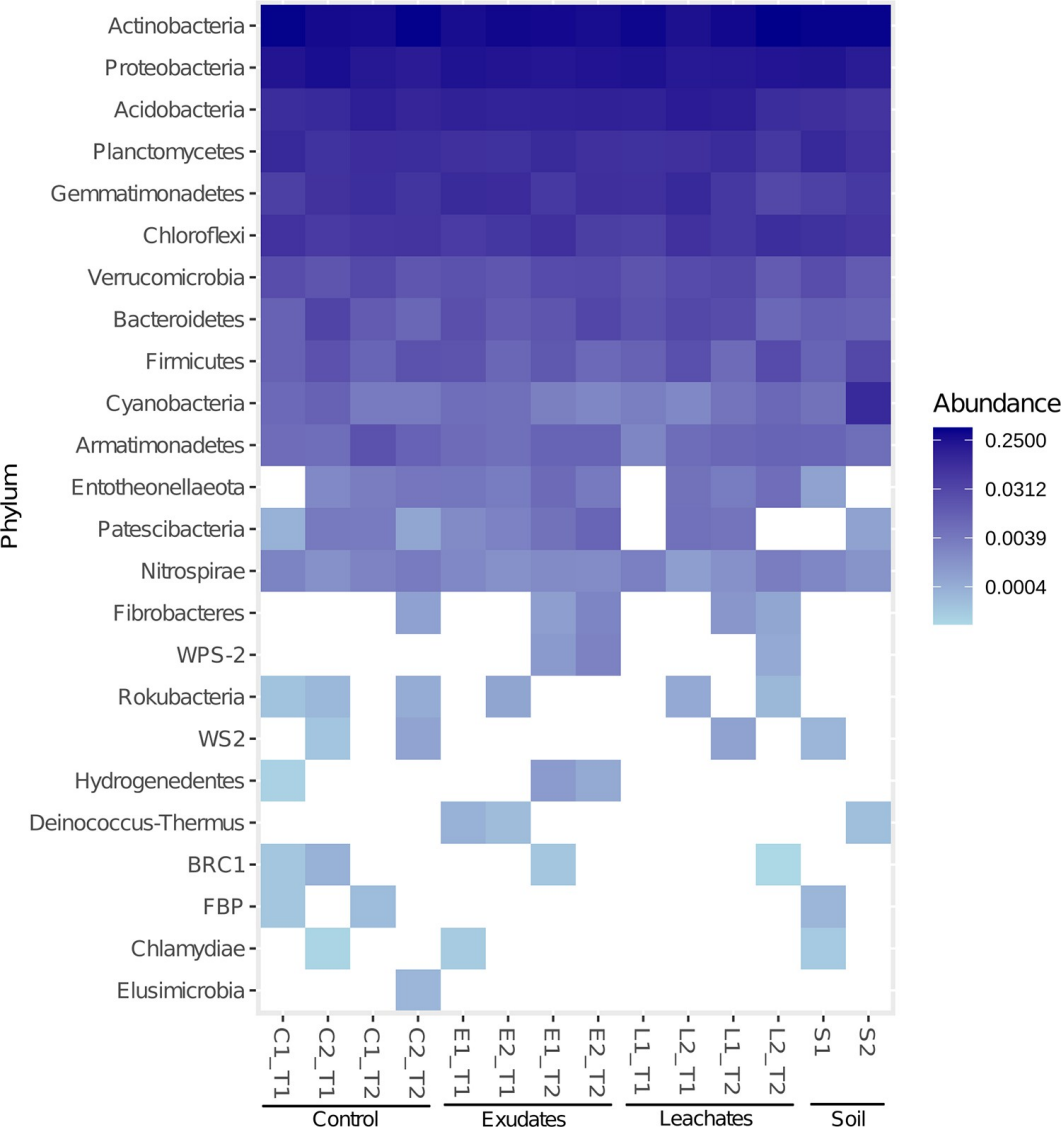
Buffelgrass microbiome at the phylum level. Each sample is labeled indicating control (C), exudates (E), leachates (L), soil (S), and sampling time (T1 and T2).

We detected 24 phyla in all our samples. Eleven phyla were ubiquitous and abundant: *Actinobacteria*, *Proteobacteria*, *Acidobacteria*, *Planctomycetes*, *Gemmatimonadetes*, *Chloroflexi*, *Verrucomicrobia*, *Bacteroidetes*, *Firmicutes*, *Cyanobacteria*, and *Armatimonadetes* (Fig 2, https://doi.org/10.6084/m9.figshare.c.6605350.v2). The heatmap also revealed differences in the microbiome composition among different periods. For example, *WPS-2* and *Hydrogenedentes*, whose abundances increased in exudates from T1 (relative abundance values of *WPS-2* = 0.00; *Hydrogenedentes* = 2.05E-05) to T2 (*WPS-2* = 7.29E-04; *Hydrogenedentes* = 2.64E-04). On the contrary, the abundance of *Deinococcus-Thermus* decreased from T1 (1.16E-04) to T2 (0.00) in the same treatment. The abundance of the phylum *Fibrobacteres* increased over time both in the exudate and the leachate treatments. Complete taxonomic annotation and abundances are available as Supplementary Material (S1 and S2 Tables, https://doi.org/10.6084/m9.figshare.c.6605350.v2).

**Fig 3:**
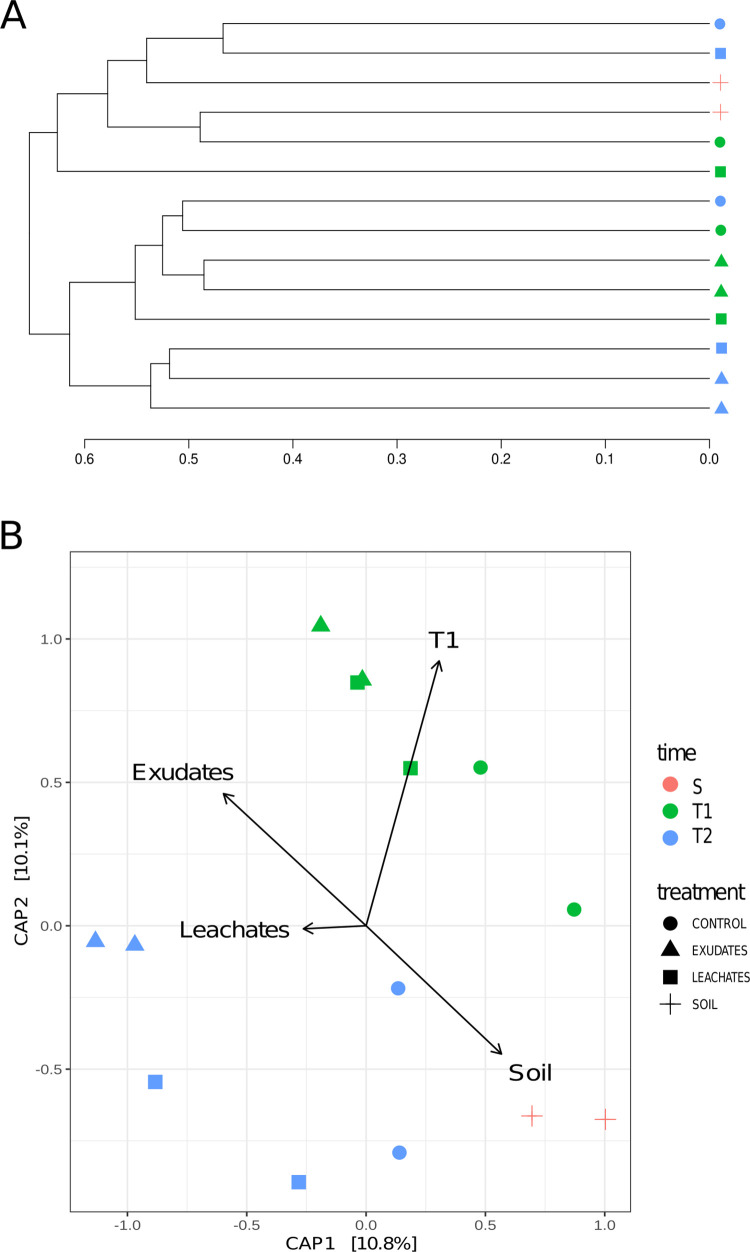
Beta diversity of the buffelgrass microbiome. (A) Dendrogram of microbiome relatedness based on an unweighted UniFrac distance matrix (A). (B) Beta diversity of the buffelgrass microbiome is represented as a constrained analysis of principal coordinates (CAP) based on an unweighted UniFrac distance matrix for all treatments and natural soils (B). Vectors display the experiment’s different treatments and time points. Statistical significance was evaluated using the ANOSIM test for treatments (*p =* 0.092) and time (*p =* 0.0366).

The UniFrac dendrogram shows most communities clustered together according to time (Fig 3A, https://doi.org/10.6084/m9.figshare.c.6605350.v2). T2 showed larger dispersion in the dendrogram, suggesting community diversification over time. We further evaluated ß-diversity through a constrained analysis of principal coordinates (CAP) based on an unweighted UniFrac distance matrix (Fig 3B). The CAP analysis explained 20.9% of the observed variance. The x-axis described most of the ordination variance (10.8%) and separated the natural soil samples from the buffelgrass samples on the bottom right side of the ordination (Fig 3B). Control samples (C) are closer to the natural soils than the samples from allelochemical treatments (E and L). The CAP ordination clusters were evaluated with ANOSIM to test the treatment differences; no significant differences were found (R: 0.06579; p = 0.2902; 9,999 permutations). All T1 samples clustered at the upper half, as opposed to T2, located in the lower half of the ordination (Fig 3B). The ANOSIM test showed that time-based clustering was statistically significant (R: 0.2258; p = 0.0423, 9,999 permutations), meaning that the samples differed depending on time.

**Fig 4:**
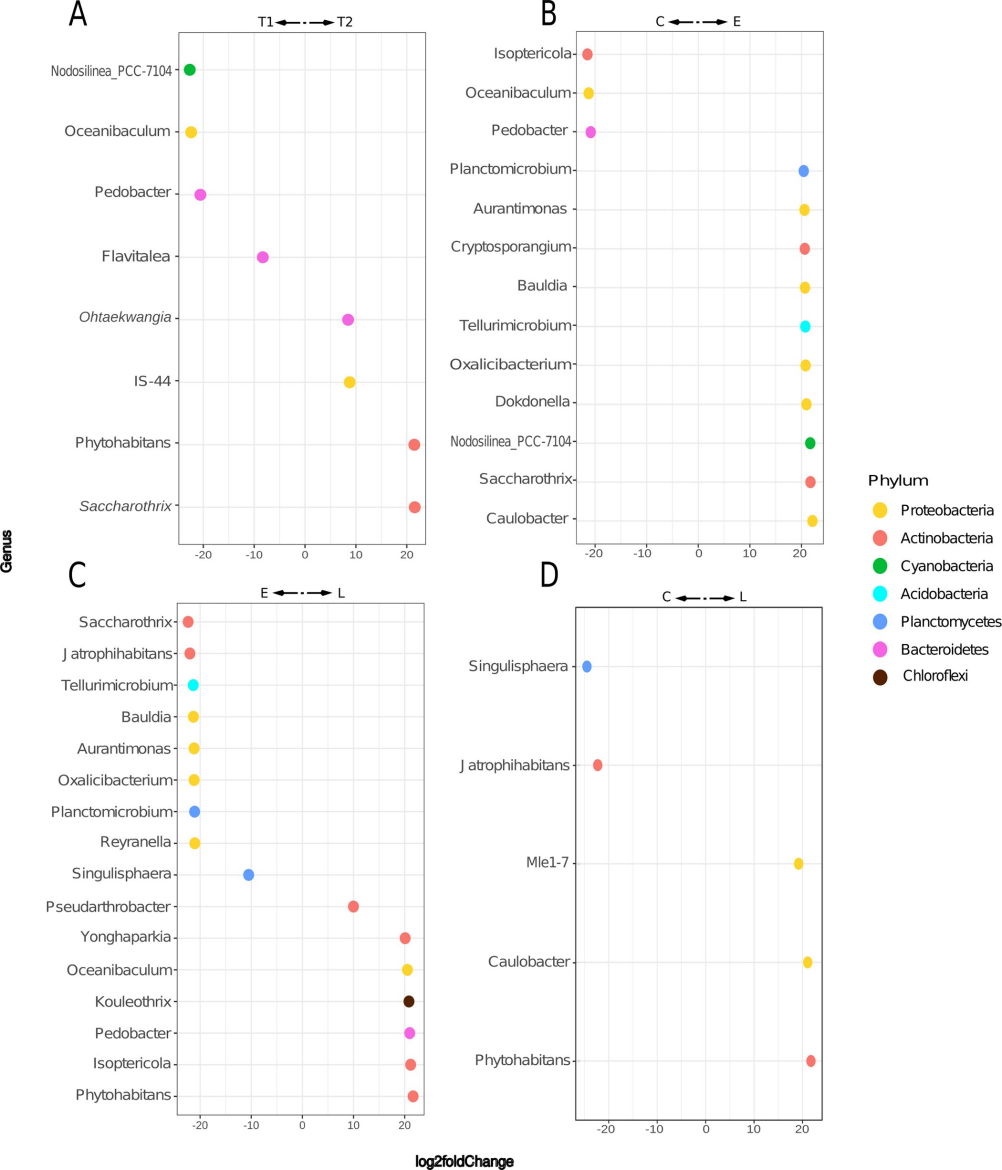
Enriched bacterial genera in allelochemical treatments. Shown are (A) the log2fold ratio between T1 and T2 (A), (B) exudates and leachates (B), (C)exudates and controls (C), and (D) leachates and controls (D). DESeq2 was used to get the significantly enriched genera in each condition using an **α** = 0.01.

We used a DESeq2 analysis to compare and identify abundant differential genera at each time point, irrespective of the treatment used (Fig 4A, S3 Table, https://doi.org/10.6084/m9.figshare.c.6605350.v2). In T1, we identified the following genera: *Nodosilinea_PCC-7104*, *Oceanibaculum*, *Pedobacter*, and *Flavitalea*, whereas *Ohtaekwangia*, *IS-44*, *Phytohabitans*, and *Saccharothrix* were enriched in T2. Subsequently, we compared the microbiome diversity at the genus level and found 14 genera representing 16 ASVs shared in all samples, including natural soils (S2 Fig, https://doi.org/10.6084/m9.figshare.c.6605350.v2). However, these genera represented only 0.78% of the whole ASVs dataset. Most ASVs were shared between all samples, with only 13 ASVs being unique to soil samples.

**Fig 5:**
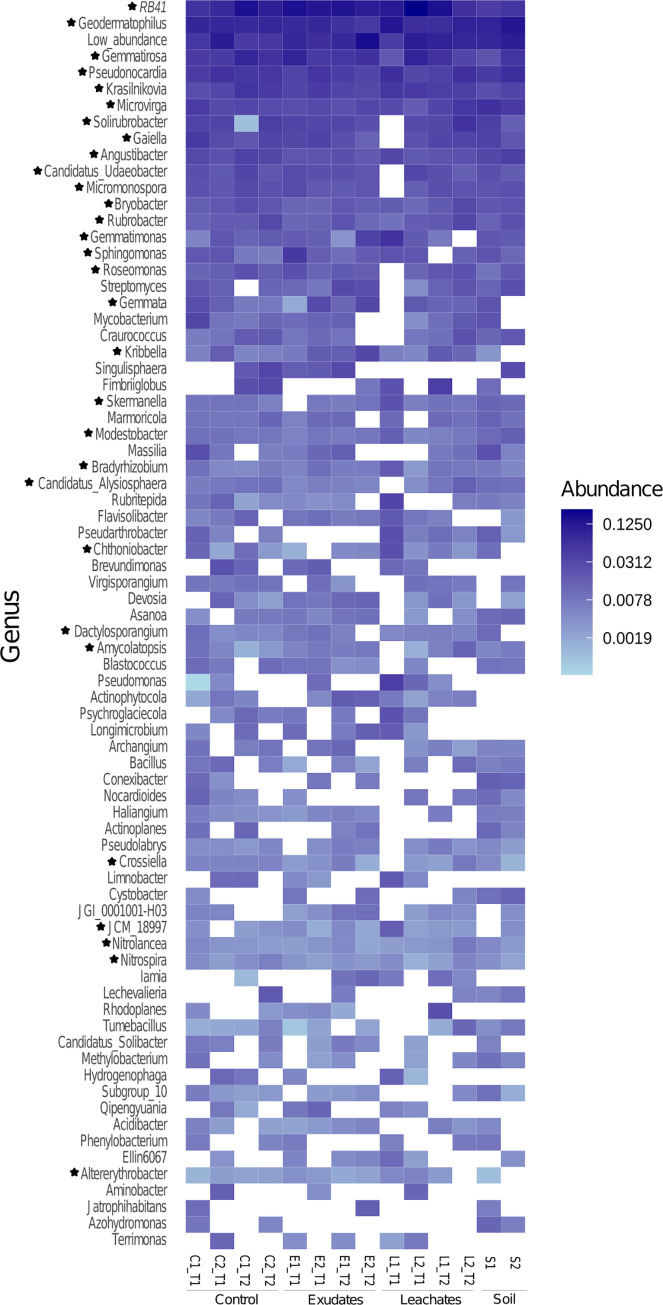
Buffelgrass core microbiome at the genus level. In the heatmap, genera marked with stars comprise the buffelgrass core microbiome. We collapsed genera with low abundances (< 0.03%).

*Actinobacteria* were highly dominant across treatments and time. We found 30 genera in the treatment and control samples, comprising the buffelgrass core microbiome (Fig 5; S1 Fig, https://doi.org/10.6084/m9.figshare.c.6605350.v2). The core genera were *Geodermatophilus*, *RB41*, *Krasilnikovia*, *Angustibacter*, *Microvirga*, *Kribbella*, *Bradyrhizobium*, *Rubrobacter*, *Modestobacter*, *Pseudonocardia*, *Gemmatirosa*, *Bryobacter*, *Nitrospira*, *Nitrolancea*, *Solirubrobacter*, *Gaiella*, Candidatus*_Udaeobacter*, *Dactylosporangium*, *Altererythrobacter*, *Micromonospora*, *Roseomonas*, *Gemmata*, *Skermanella*, *Chthoniobacter*, *Gemmatimonas*, *Sphingomonas*, *JCM_18997*, Candidatus*_Alysiosphaera*, *Amycolatopsis*, and *Crossiella* (Fig 5). Most of them belong to the phylum *Actinobacteria*, except for *RB41* (*Acidobacteria*), *Gemmatirosa* and *Gemmatimonas* (*Gemmatimonadetes*), Candidatus_*Udaeobacter* and *Chthoniobacter* (*Verrucomicrobia*), *Gemmata* (*Planctomycetes*), and *Sphingomonas*, Candidatus_*Alysiosphaera*, *Roseomonas*, *Skermanella*, *Altererythrobacter*, and *Microvirga* (*Proteobacteria*). A complete list of the core microbiome species and a summary of where they had been isolated is available in S4 Table (https://doi.org/10.6084/m9.figshare.c.6605350.v2).

We identified enriched genera in the leachates compared to the controls, namely *Mle1-7*, *Caulobacter*, and *Phytohabitans* (Fig 4D, S3 Table). Compared to the controls, the genera enriched in the exudates were *Planctomicrobium*, *Aurantimonas*, *Cryptosporangium*, *Bauldia*, *Tellurimicrobium*, *Oxalicibacterium*, *Dokdonella*, *Nodosilinea-PCC-7104*, *Saccharothrix*, and *Caulobacter* (Fig 4B, S3 Table). The following genera were higher in the exudates than the leachates: *Saccharothrix*, *Jatrophihabitans*, *Tellurimicrobium*, *Bauldia*, *Aurantimonas*, *Oxalicibacterium*, *Planctomicrobium*, *Reyranella*, and *Singulisphaera* (Fig 4C, S3 Table). The genera enriched in the leachates were *Phytohabitans*, *Isoptericola*, *Pedobacter*, *Kouleothrix*, *Oceanibaculum*, *Yonghaparkia*, and *Pseudarthrobacter* (Fig 4C, S3 Table).

## 4. Discussion

### 4.1 The buffelgrass microbiome, clues for success as an invasive species?

Exploring the microbiomes of invasive species may shed light on the establishment and propagation of those species and their impacts on ecosystems, hence providing a turning point for developing new plant control strategies. Even though a high number of ASVs are shared between all treatment and soil samples, the microbiome composition of the rhizosphere of buffelgrass is distinguishable from that of the soil microbiome (Fig 3B, S1 Fig). Our results suggest that the overall bacterial communities of *Pennisetum ciliare* are structured by different factors. Overall, the Shannon values obtained for the samples in this study were within the range of Shannon values (*H’* = 5.1811–5.5709, Table 1) reported for microbiomes in desert soils [39]. Regarding the exudate treatment, previous reports have shown that the rhizosphere microbial diversity tends to be higher when treated with root exudates, specifically when exposed to phenolic compounds [40]. These diversity observations align with the Shannon values obtained for exudates (*H’* = 5.51) and control treatments in T2 (*H’* = 5.24). The diversity for buffelgrass exposed to root exudates at the end of the experiment (*H’* = 5.51) was higher than the value from the bulk soil (*H’* = 5.34), a pattern already reported for ruderal plants, whose diversity in the rhizosphere tends to be higher than in bulk soil [41–43]. Our data showed that the rhizosphere microbiome of buffelgrass is dominated by the phyla *Actinobacteria*, *Proteobacteria*, *Acidobacteria*, *Planctomycetes*, *Gemmatimonadetes*, and *Chloroflexi* (Fig 2), in agreement with a previous report [2]. Likewise, the surrounding soil of the allelopathic desert shrub *Artemisia sieberi* shows similar phyla diversity [11]. The buffelgrass microbiome composition is also dominated by *Actinobacteria*, as reported in multiple arid areas [44, 45]. The ability of actinobacterial spores to germinate in environments with low water availability enables their adaptation to drought conditions [46] and shrub root zones of deserts [47].

Regarding the genus diversity, *RB41*, *Geodermatophilus*, *Gemmatirosa*, *Pseudonocardia*, *Krasilnikovia*, *Microvirga*, *Solirubrobacter*, *Gaiella*, *Angustibacter*, *Candidatus_Udaeobacter*, *Micromonospora*, *Bryobacter*, *Gemmatimonas*, *Sphingomonas*, and *Roseomonas* were highly abundant in buffelgrass, irrespective of the treatment or sampling time (Fig 5). Overall, 2,164 ASVs corresponded to 235 different genera of bacteria detected in our samples. Those 235 genera constitute the extended microbiome of buffelgrass roots under all tested conditions (S1 Fig). Those bacteria comprise what is referred to as the buffelgrass core microbiome. A core microbiome is a set of microorganisms forming cores of interactions that can be used to optimize microbial functions at the individual plant and ecosystem levels [23]. Diversity manipulation might be a key battleground where hosts and various hubs cooperate or compete, making them potential targets for plant biocontrol studies [48]. Rather than just promoting host plant growth, the core microbiome is vital in organizing the community assemblies mediating and organizing plant-microbe and microbe-microbe interactions by recruiting indigenous microorganisms with diverse functions and even suppressing high pathogen loads in the field [23]. Hence, the detailed study of core microbiomes should enable microbial species identification and functions on plant-microbe interactions that impact plant adaptation to arid environments and their featured plant-associated lifestyles [49, 50].

Among the core microbiome of buffelgrass (Fig 5, S2 Fig), some genera have been related to allelopathic conditions, such as *RB41*, *Bryobacter*, *Nitrospira*, *Gaiella*, and *Microvirga*, whose abundances changed depending on the amount of vanillic acid available [20, 51, 52]. Additionally, the genus *Nitrospira* has been reported in the soil surrounding buffelgrass and can oxidize ammonia to nitrate (commamox) [2]; commamox activity is frequent in oligotrophic habitats [53]. Hence, the presence of this genus in the core microbiome could help buffelgrass obtain nitrogen in deserts, where this element tends to be scarce. *Bradyrhizobium* is a common species in close association with plant roots, including known invasive species such as *Acacia dealbata*, and can enhance the competition ability of the tree [21, 54]. The potential for producing soluble vitamins, antimicrobials, and antibiotics detected in rhizosphere metagenomes and genomes of *Bradyrhizobium*, *Geodermatophilus*, *Pseudonocardia*, *Micromonospora*, *Crosiella*, *Amycolatopsis*, and *Kribbela*, may also be relevant in the context of plant invasions [55–60]. The genus *Sphingomonas* produces molecules that promote plant growth [61], whereas *Kribbella* isolates from allelopathic shrubs and other plant species show antifungal activity and contain genes related to the production of secondary metabolites [62, 63].

*Gemmatimonas* belong to the phylum *Gemmatimonadetes*, including taxa adapted to arid and oligotrophic conditions, and are among the most abundant bacteria in soils [64, 65]. The ability to thrive in replanted soils and to accumulate polyphosphates may be beneficial for the development of buffelgrass, which creates an allelopathic autotoxic environment and depends on phosphorus compounds to develop [5, 17, 66–68].

*Solirubrobacter* (*Actinobacteria*) is ubiquitous in buffelgrass. Only a few cultivated bacteria represent *Solirubrobacter*, with indirect evidence that showed them as a diverse group in nature [69]. *Solirubrobacter* can thrive in phenolic environments, using chlorogenic acid to grow [69], and can develop in the rhizosphere of cucumber treated with vanillin [20]. The relationship of *Solirubrobacter* with the plant host phenotype is ambiguous, with positive and negative correlations in multiple hosts [67, 70, 71]. Considering that several studies propose that certain fungi and bacteria contribute to the invasion success of plants introduced to novel habitats [25, 54], it is fair to suggest that the microorganisms recruited in the rhizosphere of buffelgrass contribute to a certain extent to the establishment and quick development of this grass in the habitats it invades.

### 4.2 Buffelgrass recruits taxa capable of thriving in an allelopathic environment

The allelochemicals exogenously amended during the experiment favored differential taxa abundance, according to our DESeq2 analysis (Fig 4). This effect of allelochemicals on microbiome structuring was observed in the CAP analysis, where the samples from the exudate and leachate treatments from T2 are further apart than they were in the first period (Fig 3B). Moreover, microbiomes from allelochemical treatments clustered apart from both soil and the controls. Even though the microbiomes under leachate treatment cluster closer to the controls, the distance is noticeable (Fig 3B). This strongly suggests that the presence of buffelgrass influences the root microbiome diversity.

Plants in desert ecosystems have developed ecophysiological adaptations, including salt and chemical compound excretion beneath the plant canopy, creating an allelopathic environment that affects organic matter and soil moisture levels [11]. Previously reported buffelgrass roots exudates comprised chlorogenic, ferulic, caffeic, *p*-OH-benzoic, *p*-coumaric, vanillic, syringic, and gentisic acids, all phenolic acids, and proven allelopathic agents [17]. Phenolic compounds recruit bacterial taxa more precisely than other rhizodeposits [72], and some compounds, such as coumaric acid, affect growth and microbial community composition in a concentration-dependent manner [19, 73]. Moreover, this concentration may increase through soil microbial production and using vanillic acid and *p*-OH-benzoic acid from ferulic acid and *p*-coumaric acid, respectively [19, 74, 75]. Since the buffelgrass microbiome changes across time and development, the root-recruited taxa are likely able to metabolize the phenolic compounds secreted by the roots at different growth stages [25].

We found 17 abundant differential bacterial genera in the treatments. As expected, some ASVs enriched in the exudates or leachate treatment are known for their capabilities to metabolize and produce phenolic compounds (S3 Table). Enriched taxa in allelochemical treatments were: *Caulobacter*, *Phytohabitans*, *Mle1-7*, *Planctomicrobium*, *Aurantimonas*, *Cryptosporangium*, *Bauldia*, *Tellurimicrobium*, *Oxalicibacterium*, *Dokdonella*, *Nodosilinea-PCC-7104*, and *Saccharothrix* (Fig 4B and 4D, S3 Table). *Caulobacter* (*Alphaproteobacteria*) was the only one enriched in both allelochemical treatments. *Caulobacter* acts as a microbial community hub on the leaf microbiome of *Arabidopsis* [48]; also as a plant growth-promoting bacterium (PGPB) through the production of plant hormones [76, 77]. Different species of *Caulobacter* can thrive in rhizosphere environments containing the same phenolic acids as those produced by buffelgrass [18, 78–80]. *Cryptosporangium* and *Bauldia* are found in the roots of different allelopathic plants, such as *Eucalyptus* and *Andrographis* [81, 82].

Buffelgrass is a plant species preferred for grazing because of its high nutrient content. However, it can accumulate oxalate to potentially toxic concentrations for cattle [5, 17, 83]. *Oxalicibacterium* (*Burkholderiaceae)* comprises species isolated from soils and litter from oxalate-forming plants and collects oxalic acid or calcium oxalate as metabolic products, although oxalate can also be produced through the degradation of other compounds such as citrate [84–88]. Bacterial oxidation of calcium oxalate, which can be carried out by species such as *Oxalicibacterium* and *Bradyrhizobium*, entails soil alkalinization, which may act as a soil acidification buffer [88]. Similarly, *Tellurimicrobium* can grow on oxaloacetate [89]. The relevance of the ability to metabolize oxalic compounds is rooted in the importance of oxalate regarding plant physiology. Oxalate solubilizes insoluble phosphorus (in the form of aluminum phosphate) in the rhizosphere, is involved in pH regulation and calcium storage, and acts as a ligand for aluminum [85, 90]. Furthermore, oxalate degradation by some taxa, such as *Burkholderia*, lowers oxalate levels on plant surfaces and decreases the infection potential of pathogenic microorganisms attracted by oxalate [90].

Allelochemicals are compounds synthesized not only by plants but also by fungi and bacteria. The actinobacterium *Saccharothrix* was also enriched in the root exudate treatment compared to the controls. This genus can biosynthesize caffeic acid and cinnamic acid, the latter being a substrate for the biosynthesis of other phenolic compounds [91, 92]. The biosynthesis of phenolic compounds, such as vanillic acid from ferulic acid, through mechanisms involved with ß-oxidation, has been described in many bacteria [75]. The accumulation of allelochemicals, such as phenolic compounds, may have both plant and bacterial origins. Buffelgrass is a plant with a C4 metabolism, which enables it to thrive under drought conditions and high temperatures [6, 93]. Under those conditions, plants draw upon different strategies, such as regulating stomatal aperture [94]. Albeit in a concentration-dependent manner, phenolic acids influence the aperture and closure of stomata in various plant species [95–97] and may even alter photosynthetic performance [98]. Hence allelochemicals could act as signals in a cross-kingdom talk, and it is possible that the presence of phenolic compounds, either supplied by the plant or by the microorganisms, in the buffelgrass rhizosphere influences buffelgrass physiology through the regulation of stomatal aperture and closure, which in turn could diminish the growth of the plant while promoting its development in an arid environment. Further analysis using a metagenomic perspective may confirm the interpretations made in this study.

### 4.3 Time as a microbiome community-structuring factor

Rhizosphere microbiomes are composed of bacteria filtered by the same plant from the bulk soil, creating microbial communities in the rhizosphere, distinguishable from the soil communities [99–101]. The soil-to-rhizosphere effect was shown in the ordination (10.8% of the variance, Fig 3B), where the samples from bulk soil (S) cluster apart from all the treatment samples derived from buffelgrass plants. Interestingly, the Unifrac dendrogram shows a closer phylogenetic relationship between communities belonging to the same period (Fig 3A). Similarly, CAP analysis revealed that time influences the microbiome structure, shown through differences in the microbiome between T1 and T2 (Fig 3B). Studies have reported plant developmental effects on plant-associated microbiomes [102–104]. Even though the period evaluated is relatively short (20 days difference), specific phyla changed in their abundances concerning T1 and T2 (Fig 2). The *WPS-2* and *Hydrogenedentes* showed increased quantities from T1 to T2 in the root exudates treatment. In contrast, *Fibrobacteres* rose in the root exudate and leachate treatment. *Hydrogenedentes* have been reported in contaminated zones with compounds such as phenanthrene [105] and associated with multiple plants [40,106]. Phylum *WPS-2* has been registered as dominant in the invasive tree *Eucalyptus*, with a declining abundance after the first year [107]. *Deinococcus-Thermus* abundance was reduced from T2 to T1 in the root exudate treatment; in a previous study, the absence of phenolic allelochemicals correlated with a decreased *Deinococcus-Thermus* abundance [40].

Additionally, plant age is the main factor shaping root microbiomes in maize and *Eucalyptus*, stabilizing over time [107, 108]. Regarding buffelgrass, the microbiome changes probably reflect plant aging. The plant’s different development stages, including the allelochemicals exuded by the roots, cause changes in the compounds secreted by the roots [78]. Time differences were highlighted by differential taxa in buffelgrass (Fig 4A, S3 Table). During the first period (T1), the significantly enriched bacteria belonged to the phyla *Cyanobacteria* (*Nodosilinea PCC-7104*), *Proteobacteria* (*Oceanibaculum*), and *Bacteroidetes* (*Pedobacter* and *Flavitalea*). *Pedobacter* and *Oceanibaculum* have increased abundances during the intermediate plant growth stages [109]. Bacteria in the rhizosphere of younger plants tend to use simple amino acids instead of complex carbohydrates, as do older plants [110]. This young plant hypothesis is backed by previous studies, where the amendment of soils with a mixture of long-chain fatty acids and amino acids led to an enrichment of *Flavitalea* [111].

We observed that *Bacteroidetes* (*Ohtaekwangia*), *Proteobacteria* (*IS-44*), and *Actinobacteria* (*Phytohabitans* and *Saccharothrix*) were enriched in T2. This is consistent with previous findings that *Ohtaekwangia* dominates in the middle-growth stages of other plant microbiomes and decreases over time [104, 109]. Our results also agree with this pattern, as T2 buffelgrass plants were not at a young growth stage, and *Ohtaekwangia* was enriched in 112-day-old buffelgrass plants.

The changes in root exudates of a plant throughout its development can significantly impact the microbial communities in the surrounding soil, as highlighted by recent studies [78, 112]. This highlights the importance of considering the plant’s phenology and physiological and chemical traits when examining the effects of invasive plant species on ecosystems [1, 73]. Additionally, the impact of phenolic compounds on the soil microbiome is known to be concentration-dependent [19, 73], and changes in root exudate patterns during the development of buffelgrass could provide new insights into the formation of rhizosphere microbiomes. Our experimental design, which included the use of PVC pots, may have also played a role in the accumulation of phenolics and leachates, leading to the selection of microorganisms that are capable of thriving in environments with high concentrations of phenolic compounds or can degrade these compounds as part of their metabolism. These microorganisms are probably selected based on the buffelgrass’s growth stage and allelochemicals’ fate and concentration. This interaction could allow the plant to benefit from the production of antimicrobial compounds and the degradation of contaminants, allowing the plant to expand its invasive range.

## 5. Conclusions

*Actinobacteria* dominated the microbiome of buffelgrass, resembling the microbiomes of other plants in deserts, arid zones, and other desert allelopathic shrubs. Our study found that rhizosphere microbiomes treated with allelochemicals had a higher abundance of specific taxa, highlighting the impact of buffelgrass on soil bacteria. Time was also found to be a significant factor in shaping the buffelgrass microbiome, and further research should focus on the effects of allelochemicals on the root exudate pattern throughout the plant’s development. Additionally, we identified a core microbiome comprising microorganisms known for their antimicrobial or vitamin production capabilities, which likely played a role in shaping the remaining microbial community associated with the roots of buffelgrass. Our findings suggest that certain recruited bacteria can metabolize allelochemical compounds excreted by the roots, potentially influencing buffelgrass physiology.

## Supporting information

S1 Fig Buffelgrass microbiome composition heatmap at the genus level. (EPS)

S2 Fig Shared genera between samples visualized in an UpSet plot. Shared taxa at the genus level between allelochemical and control treatments. The histogram shows the number of shared elements for each intersection set, ordered in a decreasing manner. Genera marked with stars comprise the buffelgrass core microbiome, considering taxa present in all treatment and control samples but allowing absence in one of the samples.
(TIFF)

S1 Table Taxonomy table indicating the taxonomic assignment of each ASV. (XLSX)

S2 Table Counts table indicating the number of appearances of each ASV per sample. (XLSX)

S3 Table Genera significantly enriched in paired comparisons. The table shows significantly differentially abundant bacteria calculated by DESeq2 analysis. Information about the microbe genera, sources of isolation, and associated publications are also provided.
(XLSX)

S4 Table Genera comprising the buffelgrass core microbiome. Bibliographical information about microbial sources of isolation and associated publications.
(XLSX)

S1 Dataset Phylogenetic tree. Newick file for the phylogenetic tree constructed with FastTreeMP using 16S rRNA gene sequences obtained from buffelgrass microbiome samples.
(NWK)
