# Supplementary figures and images for: Root microbiome diversity and structure of the Sonoran desert buffelgrass (Pennisetum ciliare L.)
Source: PLoS One. 2023 May 19;18(5):e0285978. doi: 10.1371/journal.pone.0285978 (PMC10198571; doi:10.1371/journal.pone.0285978)

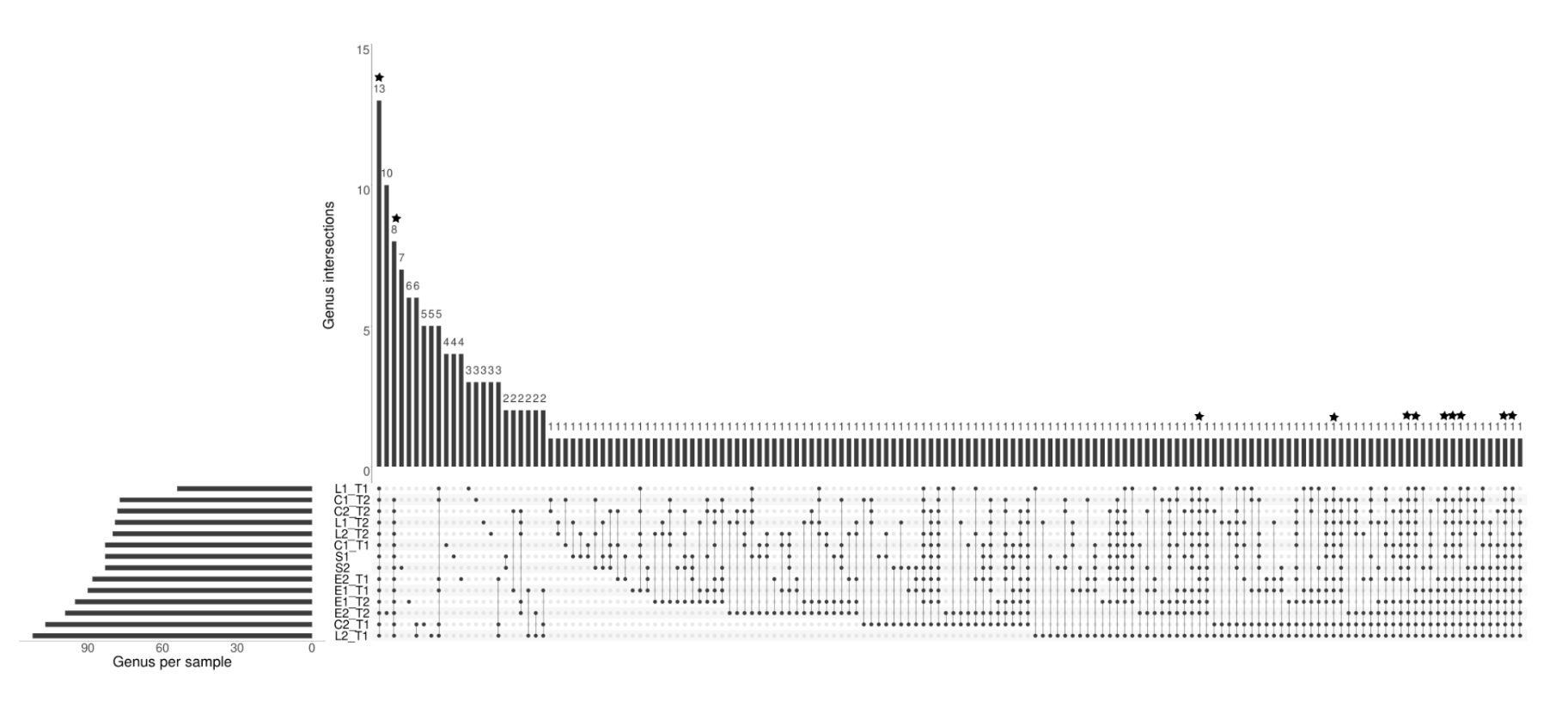

Supplement: S2 Fig — Shared taxa at the genus level between allelochemical and control treatments. The histogram shows the number of shared elements for each intersection set, ordered in a decreasing manner. Genera marked with stars comprise the buffelgrass core microbiome, considering taxa present in all treatment and control samples but allowing absence in one of the samples. (TIFF) [file pone.0285978.s002.tiff]
